# Deep intronic *GPR143* mutation in a Japanese family with ocular albinism

**DOI:** 10.1038/srep11334

**Published:** 2015-06-10

**Authors:** Takuya Naruto, Nobuhiko Okamoto, Kiyoshi Masuda, Takao Endo, Yoshikazu Hatsukawa, Tomohiro Kohmoto, Issei Imoto

**Affiliations:** 1 Department of Stress Science, Institute of Biomedical Sciences, Tokushima University Graduate School, Tokushima 770-8503, Japan; 2 Department of Medical Genetics, Osaka Medical Center and Research Institute for Maternal and Child Health, Izumi 594-1101, Japan; 3 Department of Human Genetics, Institute of Biomedical Sciences, Tokushima University Graduate School, Tokushima 770-8503, Japan; 4 Department of Ophthalmology, Osaka University Graduate School of Medicine, Suita 565-0871, Japan; 5 Eye Department, Osaka Medical Center and Research Institute for Maternal and Child Health, Izumi 594-1101, Japan; 6 Student Lab, Tokushima University Faculty of Medicine, Tokushima 770-8503, Japan

## Abstract

Deep intronic mutations are often ignored as possible causes of human disease. Using whole-exome sequencing, we analysed genomic DNAs of a Japanese family with two male siblings affected by ocular albinism and congenital nystagmus. Although mutations or copy number alterations of coding regions were not identified in candidate genes, the novel intronic mutation c.659-131 T > G within GPR143 intron 5 was identified as hemizygous in affected siblings and as heterozygous in the unaffected mother. This mutation was predicted to create a cryptic splice donor site within intron 5 and activate a cryptic acceptor site at 41nt upstream, causing the insertion into the coding sequence of an out-of-frame 41-bp pseudoexon with a premature stop codon in the aberrant transcript, which was confirmed by minigene experiments. This result expands the mutational spectrum of *GPR143* and suggests the utility of next-generation sequencing integrated with *in silico* and experimental analyses for improving the molecular diagnosis of this disease.

Ocular albinism type 1 (OA1, MIM: #300500) is the most common cause of ocular albinism, with an estimated prevalence of approximately1:50,000; it is inherited as an X-linked trait^1^. In affected men, congenital nystagmus and papillary reflex or photophobia are the main characteristics of ocular albinism^2^,^3^. The fundus is depigmented and the choroidal vessels are prominent. To a lesser extent, impaired visual activity, iris translucency, foveal hypoplasia and loss of stereoscopic vision due to reduction of the ipsilateral component of the optic tract may also occur. Pigmentation is normal in skin and hair. OA1 is caused by mutations in the gene encoding the G protein-coupled receptor 143 (*GPR143*; Gene ID: 4935, OMIM: #300808), which is located on chromosome Xp22.3, spans approximately 40 Kb of genomic DNA and comprises nine exons^4^,^5^.

According to the Human Gene Mutation Database (HGMD) (professional 2014.3; http://www.hgmd.cf.ac.uk/ac/index.php), there are more than 110 mutations in the *GPR143* gene. The vast majority are missense and nonsense mutations, and small intragenic deletions and insertions, splice-site mutations and gross deletions also occur, indicating that the disease is caused by loss of gene function. Notably, a significant percentage of patients with X-linked ocular albinism lack a detectable mutation in *GPR143*, and various approaches, including analyses of untranslated regions (UTRs), have increased the rate of detection of mutations^1^,^5^,^6^,^7^,^8^,^9^. However, the presence of undetected mutations by a standard mutational screening for the coding exons (the mutation detection rate is 35−90% of X-linked ocular albinism or OA1) represents a biological and diagnostic problem^1^,^5^,^6^,^7^,^8^,^9^.

There is no obvious explanation for this low rate of detecting mutations. The possibility that mutated genes other than *GPR143* are responsible for OA1 seems unlikely, because linkage analysis of families without *GPR143* mutations point to the OA1 locus (Xp22.3)^5^,^10^,^11^,^12^,^13^,^14^. Another possibility is that*GPR143* mutations occur outside the coding region or include larger insertions or deletions. Most intronic mutations occur within or very close to conserved splice sites, and are readily detected using Sanger sequencing of polymerase chain reaction (PCR) amplicons generated from exons. We are aware of only one deep intronic mutation (intron 7) of *GPR143*^15^. This mutation creates an aberrantly spliced mRNA by generating a *de novo* splicing enhancer motif that is similar to mutations detected by analysis of mRNAs of other genes^16^,^17^,^18^. However, genes expressed by specific cell lineages that are difficult to isolate, including pigment cells that express *GPR143*^19^, are difficult to identify through analysis of mRNAs.

Here we applied whole-exome sequencing (WES) to detect mutations and copy number variations to study one Japanese family with two male siblings affected by nystagmus and ocular albinism. Although mutations in coding regions nor copy number alterations of *GPR143* or other candidate genes were not detected, detailed analysis of intronic sequences flanking target exons presented here show that ocular albinism in this family may be caused by the deep intronic mutation c.659-131 T > G in *GPR143* (NM_000273.2) intron 5 that creates a cryptic donor splice site. Moreover, this mutation causes a 41-bp insertion in the transcript by creating a cryptic exon within intron 5.

## Results

### Clinical description

**Figure 1:**
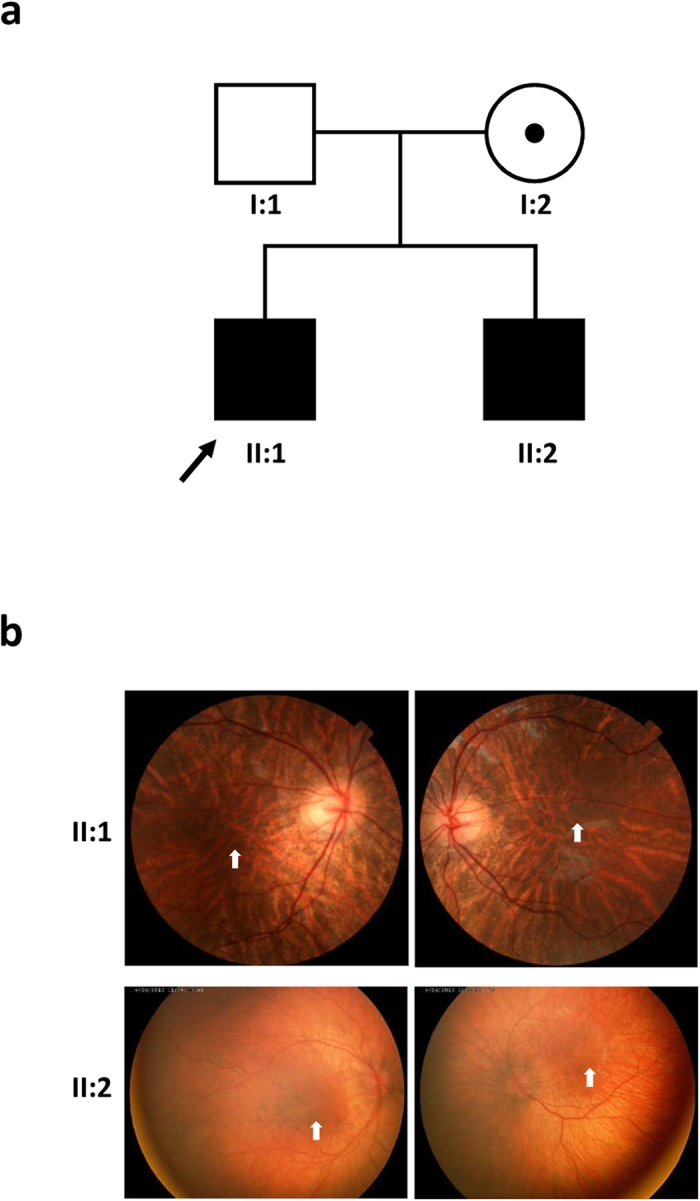
A Japanese family with OA and congenital nystagmus. (**a**) Pedigree. Black symbols indicate patients with OA. The dot represents a carrier and the probands are marked by arrows. (**b**) Representative images of fundi from patients. Fundi of both eyes of II:1 (upper) and II:2 (lower) revealed severe hypopigmentation and foveal hypoplasia (white arrows).

The proband (II:1) was a 7-year-old boy who visited our hospital because of congenital nystagmus since birth, with poor visual activity and tilting of the head to the right (Table 1). Therefore, we recommended that he wear glasses to correct astigmatism. He is the first child of non-consanguineous parents. Ophthalmological examination revealed hypopigmentation of the fundus and macular hypoplasia in both eyes, but his irises were normal (Fig. 1, Table 1). His skin and hair, as well as his physical growth and development, were normal.

II:2 is the younger brother (23 months) of II:1. He exhibited congenital nystagmus since birth, but his visual acuity was not affected. Ophthalmological examination revealed hypopigmentation of the fundus and macular hypoplasia in both eyes (Fig. 1, Table 1). His iris, skin and hair were normal as were his physical growth and development.

### Genetic analyses

Patients were diagnosed with ocular albinism according to clinical findings and family history. At least two general forms of ocular albinism are distinguished as follows: X-linked ocular albinism (XLOA, OA1 and 2) and so-called autosomal recessive ocular albinism (AROA) or oculocutaneous albinism (OCA, OCA1–7). Genes that may cause these diseases include the following: *GPR143* (OA1), *CACNA1F* (OA2 or Aland Island eye disease, OMIM #300600), *TYR* (OCA1A and 1B, OMIM #203100 and #606952, respectively), *OCA2* (OCA2, OMIM #203200), *TYRP1* (OCA3, OMIM #203290), *SLC45A2* (OCA4, OMIM #606574), *SLC24A5* (OCA6, OMIM #113750) and *C10orf11* (OCA7, OMIM #615179). We employed a next-generation sequencing system to perform whole-exome analysis of these genes using genomic DNAs of the parents and the two affected male siblings.

**Figure 2:**
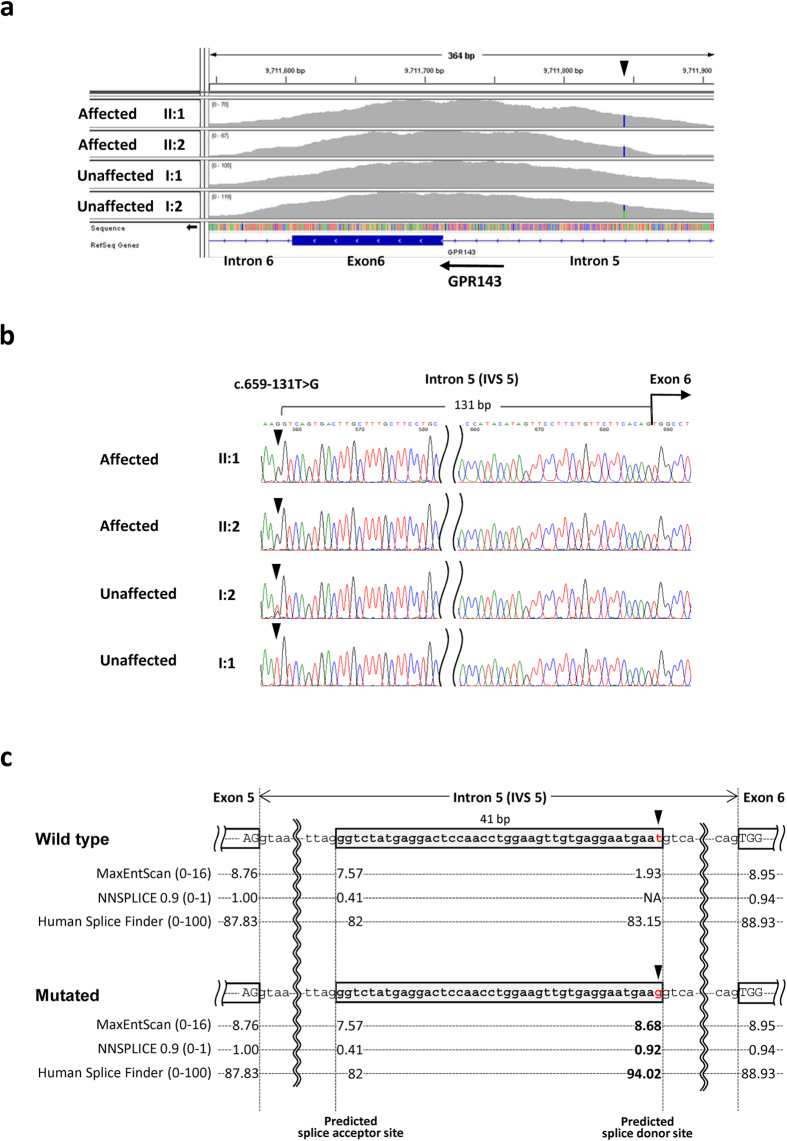
Deep intronic mutation within *GPR143* intron 5. (**a**) WES coverage data around *GPR143* exon 6 of the SNV calls of interest of the family members shown using the Integrated Genomics Viewer. The arrowhead denotes mutated bases and the arrow indicates the direction of transcription.(**b**) Sanger sequence analysis of *GPR143* intron 5 and exon 6. Arrowheads denote the mutated base. Electropherogram of the affected siblings (II:1 and II:2) showing a hemizygous c.659-131 T > G *GPR143* mutation. Electropherogram of their mother’s (I:2) sequence showing the heterozygous c.659-131 T > G *GPR143* mutation that was undetectable in their father’s (I:1) DNA.(**c**) Predictions of splice sites in the wild-type and mutated *GPR143* genomic sequences. Diagram of the *GPR143* region comprising exons 5 and 6. Closed arrow indicates intron 5 (IVS5). Arrowheads indicate the mutated base. Gray boxed sequences represent the 41-bp pseudoexon created by the mutation. The scores calculated using MaxEntScan, NNSPLICE and Human Splicing Finder are displayed below each splice site. A higher score predicts a strong splice site. The values provided in parentheses indicate the score ranges in each algorism. Note that scores of a potential cryptic splice donor site created by the mutation was almost the same levels as those of authentic splice donor and acceptor sites for IVS5.

Candidate mutations within the coding sequences and exon–intron boundaries corresponding to an autosomal recessive model, including compound heterozygosity and X-linked recessive models, are shown in Table S1. Although we evaluated the pathogenic significance of each variant, we did not detect mutations in genes possibly responsible for ocular albinism. Furthermore, we searched for mutations within flanking intronic sequences of targeted coding exons of candidate genes, which were obtained using WES, to identify pathogenic variants within non-coding sequences including introns, UTRs and regulatory regions. Through this analysis, we identified one hemizygous mutation NM_000273.2:c.659-131 T > G (NC_000023.10:g.9711844 A > C) within *GPR143* intron 5 in both affected individuals (Fig. 2a). This mutation was confirmed using Sanger sequencing, and the heterozygous *GPR143* c.659-131 T > G mutation was detected in their mother’s DNA (Fig. 2b). This mutation is not present in dbSNP138, 1000 Genomes Project, ESP6500, and HGVD. We searched for potential splice sites using three different algorithms, such as MaxEntScan, NNSPLICE 0.9 and Human Splice Finder Version 2.4.1, and found that the mutation created a potential cryptic splice donor site and an out-of-frame 41-bp pseudoexon that generates a frameshift (Fig. 2c). Therefore, we speculated that the *GPR143* c.659-131 T > G mutation affects the splicing of the *GPR143* primary transcript and may cause ocular albinism. The mutation is not recorded in databases of genetic mutations responsible for human inherited diseases, such as the HGMD, ClinVar (http://www.ncbi.nlm.nih.gov/clinvar/), and the Locus Specific Mutation Databases of the Human Genome Variation Society (http://www.hgvs.org/locus-specific-mutation-databases/).

### Minigene assay

**Figure 3:**
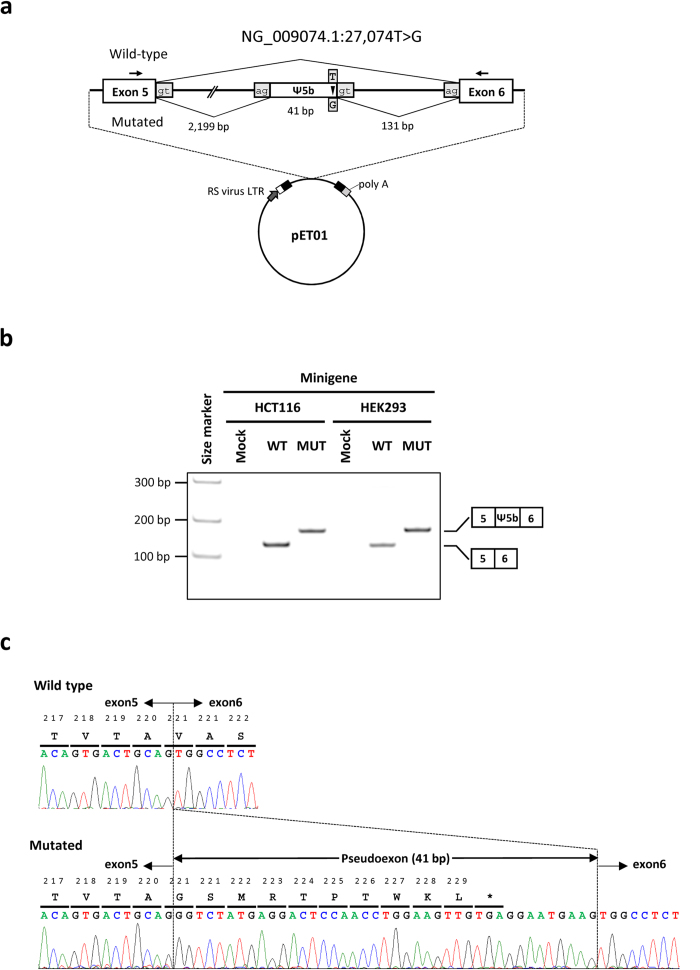
Minigene analysis of the effect of the c.659-131 T > G mutation on *GPR143* pre-mRNA splicing. (**a**) Diagrams of intron 5 and flanking exons 5 and 6 where the deep cryptic mutation c.659-131 T > G is located (upper) and the pET01-INT5 construct (lower). In the pET01-INT5 construct, the intron containing the multiple cloning site (MCS) is framed by the 5’-donor and 3’-acceptor splice sites of the preproinsulin exon (http://www.mobitec.com/cms/products/bio/04_vector_sys/exontrap.html). The expression of this vector sequence is driven by the promoter present in the long terminal repeat (LTR) of Rous Sarcoma Virus followed by a short stretch of a eukaryotic gene (phosphatase). The mutation detected in *GPR143* intron 5 (arrowhead) creates a cryptic donor splice site and generates a pseudoexon (Ψ5b) of 41 bp in the mRNA. Primers used for RT-PCR experiments are indicated by the arrows. The length of each fragment is also indicated. (**b**) RT-PCR analysis of the correct 127-bp amplicon when HEK293 and HCT116 cells were transfected with the wild-type (WT) pET01-INT5-WT minigene. The 168-bp transcript, including cryptic exon (Ψ5b), was mainly detected in HEK293 and HCT116 cells transfected with the mutated minigene (pET01-INT5-MUT, MUT) as schematically indicated to the right of the gel. No product was detected in cells transfected with the empty pET01 vector (Mock).(**c**) Nucleotide and amino acid sequences. An arrow denotes an exon–exon boundary. Sequence analysis identified a 41-bp pseudoexon between *GPR143* exons 5 and 6 of the affected individuals with the c.659-131 T > G mutation. This pseudoexon introduces a premature termination codon after 10 amino acid residues. Sequence analysis of an unaffected individual with the wild-type allele indicated normal splicing of exons 5 and 6.

Because *GPR143* is exclusively expressed by pigment cells^19^, e.g. melanocytes from skin that were not available from each affected patient, we performed a minigene assay to analyse the effect of the c.659-131 T > G mutation on the splicing of *GPR143* transcript (Fig. 3a)^20^. Reverse transcription-PCR (RT-PCR) of total RNA obtained from HEK293 and HCT116 cells that do not express *GPR143*, which were transfected with the wild-type minigene (pET01-INT5-WT), produced a 127-bp band consistent with correct mRNA splicing (Fig. 3b). In contrast, RT-PCR analysis mainly detected a 168-bp band in cells transfected with the minigene construct containing the c.659-131 T > G mutation (pET01-INT5-MUT) (Fig. 3b). Sequence analysis of these RT-PCR products revealed that the larger fragment observed in cells transfected with pET01-INT5-MUT contained an insertion of 41 bp between exons 5 and 6 due to the activation of the downstream predicted cryptic donor splice site. The insertion of the intronic 41-bp sequence would create a frameshift followed by a premature stop at codon 221 of the mutant protein, preceded by a mutant sequence of 10 amino acid residues (p.V221Gfs10*, Fig. 3c). These findings strongly support the conclusion that the intronic mutation identified in *GPR143* has pathogenic effects.

## Discussion

Approximately 10% of mutations in disease-associated genes listed in the HGMD affect splicing, and most occur within or close to conserved consensus splice sites and are readily detected by Sanger sequencing of PCR amplicons targeting coding exons. However, deep intronic mutations associated with aberrant splicing of target genes are detected with increasing frequency in the genomes of patients with diseases, such as HPRT deficiency, Usher syndrome type 2, cystic fibrosis and megaloencephalic leukoencephalopathy with subcortical cysts type 1^21^,^22^,^23^,^24^. Evaluating such splicing defects of *GPR143* may be important for patients with OA1 because *GPR143* mutations are not present in all patients with OA1. Mutations in *GPR143* that affect splicing are present mainly in authentic splice sites (HGMD and ClinVar). Only one deep intronic mutation, which generates a consensus binding motif for the splicing factor enhancer and activates a cryptic donor-splice site that inserts an intronic fragment as a cryptic exon in the transcript, was identified through analysis of transcripts expressed by skin melanocytes obtained from patients with OA1^15^. In the present study, we report a novel intronic mutation that creates a putative cryptic splice donor site in *GPR143*. Although it was not possible to detect aberrant transcripts of *GPR143* because expression of this gene is limited to pigmented cells^19^ not available from the affected patients, *in silico* analysis and the minigene experiment demonstrated that the c.659-131 T > G mutation creates a cryptic splice that generates an aberrant transcript containing a 41-bp insertion with a premature stop codon.

Ocular albinism encompasses a group of genetic disorders, in which reduced eye pigmentation is associated with decreased visual acuity, nystagmus, strabismus and photophobia. Although OA1 or Nettleship-Falls type ocular albinism, is the most common form of ocular albinism, these symptoms are not exclusive to this disease. Moreover, the diagnosis of OA1 in black or East Asian patients in contrast to Caucasian patients is difficult; this is because former patients with OA1 often have brown irises with little or no translucency and varying degrees of fundus hypopigmentation (non-albinotic fundus)^25^,^26^.

Here the two male siblings were affected by ocular albinism and congenital nystagmus, suggesting that X-linked (XLOA) or autosomal recessive (AROA) diseases caused by mutations should be considered. Furthermore, exome sequence analysis detects genomic copy number abnormalities that cause loss-of-function, although only enriched regions of interest can be evaluated^27^. Therefore, we applied WES to analyse this family because the mutations in *GPR143* associated with OA1 are highly heterogeneous. Because a significant portion of the reads acquired using exome sequencing originate outside of the designed target regions^28^, exome data provide information useful for identifying mutations in the sequences around the target exons. For example, 27% and 24% of the single nucleotide polymorphisms (SNPs) identified using exome sequencing occur in the flanking ( ≤200 bp) sequences or in sequences >200 bp away from the target regions^29^, respectively.

**Figure 4:**
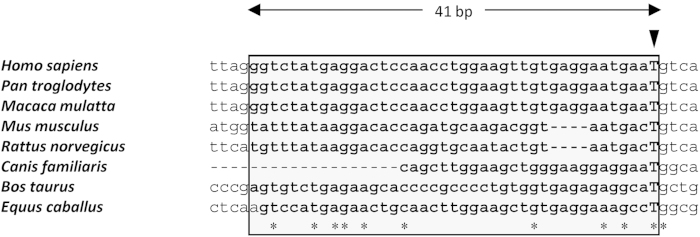
Multiple alignment of the *GPR143* intron-5 sequence around the deep cryptic mutation c.659-131 T > G site. The arrowhead indicates the mutated base. Boxed sequences represent the 41-bp pseudoexon created by the mutation. Asterisks indicate the highly conserved sites among species.

The unusual feature of the *GPR143* mutation described here is that it generates a new donor splice site within intron 5 and activates a cryptic acceptor splice site 41-bp upstream. Although the sequence c.659-131 T is evolutionarily conserved in mammals, such as *Pan troglodytes*, *Macaca mulatta*, *Mus musculus*, *Rattus norvegicus*, *Canis familiaris*, *Bos taurus* and *Equus caballus*, compared with *Homo sapiens*, the sequence around the cryptic acceptor splice site 41-bp upstream is conserved only in *P. troglodytes* and *M. mulatta* (Fig. 4). Therefore, it is unlikely that the mutation occurred around an evolutionary remnant or a ‘leftover’ of an exon within intron 5, although two of three splicing prediction algorithms yielded a relatively high score for the cryptic acceptor splice site in the human genome (Fig. 2c). Because the sequence around the mutation is not so highly conserved, it is also unlikely that the mutation occurred within consensus sequences of intronic *cis*-regulatory elements to direct transcription levels, such as enhancers, silencers, and insulators.

One limitation of this study is that the endogenous aberrant transcript containing the 41-bp insertion of the cryptic exon was not investigated in RNA samples from the two affected patients. Analyses of *GPR143* and its mRNA in patients were hampered by the restricted expression of *GPR143* in pigmented cells of the retinal pigmented epithelium and skin^19^, and such samples were unavailable from affected siblings and their mother. Therefore, we were unable to establish whether the phenotypes of our patients were most likely explained by the detected *GPR143* intronic mutation. Vetrini *et al.*^15^ analysed a pure culture of melanocytes derived from skin biopsies of patients with OA1 and detected an endogenous, aberrantly spliced mRNA with an anomalous cryptic exon, which was expressed from an intronic sequence, because a point mutation generated a consensus binding motif for the splicing factor enhancer. In their analysis, the quantities of the mutant mRNA and protein were very low, possibly due to nonsense-mediated mRNA decay caused by the premature translation-termination codon in the mutant mRNA^30^,^31^. Therefore, it may be difficult to detect expression of endogenous aberrant transcripts to diagnose OA1, even if samples suitable for expression analysis of GPR143 are available. There is the possibility that a significant number of non-exonic *GPR143* mutations contributes to OA1^1^,^5^,^6^,^7^,^8^,^9^. Therefore, integrated analyses using bioinformatics tools and experiments using cells transfected with genomic DNA fragments may determine the functional significance of mutations and facilitate early diagnosis, appropriate early therapy and genetic counselling for this OA1.

In summary, we used data obtained by WES to identify a novel deep intronic mutation c.659-131 T > G in *GPR143* in a Japanese family with male siblings affected by ocular albinism and congenital nystagmus. This mutation was predicted to create a cryptic splice donor site and an out-of-frame 41-bp pseudoexon, which creates a frameshift and a premature stop codon in the transcript, within intron 5. This *in silico* prediction was confirmed by the minigene experiments. To the best of our knowledge, this is the first report of an intronic mutation that creates a cryptic splice-donor site in *GPR143* of patients with OA1, expanding the spectrum of *GPR143* mutations. This study further illustrates the value of conducting WES analysis integrated with *in silico* and experimental analyses for molecular diagnosis of this disease.

## Methods

### Subjects

The Japanese family with two male siblings affected by congenital nystagmus (Fig. 1, Table 1) was identified and enrolled at the Eye Department of Osaka Medical Center and Research Institute for Maternal and Child Health. Clinical and ophthalmological examinations were performed in the hospital. Peripheral blood was collected from the two siblings and their parents. This study complied with the tenets of the Declaration of Helsinki. The Ethics Committee of Osaka Medical Center and Research Institute for Maternal and Child Health (Osaka, Japan) and Tokushima University (Tokushima, Japan) approved this study and informed consent was obtained from the parents before testing commenced.

### WES and Sanger Sequencing

Genomic DNA was extracted from peripheral blood leukocytes using standard methods, enriched using the SureSelect Human All Exon Kit V5 (Agilent Technologies, Santa Clara, CA) and sequenced using a HiSeq 1000 platform (Illumina, San Diego, CA) with 101-bp paired-end reads. Image analysis and base calling were performed using sequence-control software real-time analysis (Illumina) and CASAVA software v1.8 (Illumina). Reads were aligned to the human genome sequence assembly hg19 (GRCh37) using the Burrows–Wheeler Alignment tool (BWA) 0.7.10. (http://bio-bwa.sourceforge.net/). The alignments were converted from a sequence alignment map (SAM) format to sorted and indexed binary alignment map (BAM) files (SAMtools version 0.1.19). Duplicate reads were removed using Picard 1.118 (http://broadinstitute.github.io/picard/). Local realignments around insertions or deletions (indels) and base quality score recalibration were performed using the Genome Analysis Toolkit (GATK) 3.2−2 (https://www.broadinstitute.org/gatk/). Single nucleotide variants and small indels were identified using the GATK UnifiedGenotyper and filtered according to the Broad Institute’s best-practice guidelines (https://www.broadinstitute.org/gatk/guide/best-practices). Variants that passed the filters were annotated using ANNOVAR (http://www.openbioinformatics.org/annovar/). After merging the VCF files of all members of the family, we filtered variants with MAF > 0.01 from the 1000 Genomes Project databases (http://www.1000genomes.org/, 1000g2012April_all version), NHLBI GO Exome Sequencing Project (ESP6500, http://evs.gs.washington.edu/EVS/, esp6500si_all version) and the Human Genetic Variation Database (HGVD, http://www.genome.med.kyoto-u.ac.jp/SnpDB/). A summary of WES performance is shown in Table S2. To complement the SNV and indel analyses, an algorithm that detects copy number alteration (CNA) was applied to the alignment map files to identify large CNAs as follows: 1) BAM files were converted into files covering target regions using GATK, 2) log coverage ratios and Z-scores were calculated to compare the case samples with others and 3) regions with CNAs were detected using circular binary segmentation with DNAcopy (R/Bioconductor)^32^. PCR and direct Sanger sequencing using a BigDye Terminator v3.1 Cycle Sequencing Kit (Applied Biosystems, Foster City, CA, USA) were conducted with a 3130 Genetic Analyzer (Applied Biosystems) and each variant was identified in the sequences of both strands (Table S3).

Computer-assisted splice-site prediction was accomplished using MaxEntScan (http://genes.mit.edu/burgelab/maxent/Xmaxentscan_scoreseq.html), NNSpice 0.9 version (http://www.fruitfly.org/seq_tools/splice.html) and Human Splicing Finder Version 2.4.1 (http://www.umd.be/HSF/).

### *In vitro* Minigene assay

We amplified a 3,318-bp genomic DNA fragments by PCR, including intron 5 and flanking sequences of exons 5 and 6 of *GPR143* from subject II:1 carrying the hemizygous G variant and his healthy father I:1 carrying the wild-type T. PCR primers containing the appropriate restriction enzyme sites were used to amplify these DNAs (Table S3). PCR products were cloned into an Exontrap Cloning Vector pET01 (MoBiTec GmbH, Göttingen Germany) for the minigene assay. The sequences of wild-type (pET01-INT5-WT) and mutant (pET01-INT5-MUT) plasmids were validated using Sanger sequencing.

The wild-type and mutant transcripts were produced by transient transfection of HCT116 or HEK293 cells with each plasmid using the X-tremeGENE HP DNA Transfection Reagent (Roche, USA) according to the manufacturer’s instructions. Total RNA was extracted from cells 48 h after transfection using an RNAiso Plus kit (Takara, Otsu, Japan) and reverse-transcribed using ReverTra Ace reverse transcriptase (Toyobo, Osaka, Japan). Aberrantly spliced transcripts were identified by sequencing their RT-PCR products (Table S3) that were isolated after electrophoresis through 1.5% agarose gels.

## Additional Information

**How to cite this article**: Naruto, T. *et al.* Deep intronic *GPR143* mutation in a Japanese family with ocular albinism. *Sci. Rep.* **5**, 11334; doi: 10.1038/srep11334 (2015).

## Supplementary Material

Supplementary Information

## Figures and Tables

**Table 1:** **Summary of clinical features of affected males.**

**Subject**	**Sex**	**Age**	**Age of onset**	**Visual acuity (left/right)**	**Nystagmus**	**Abnormal head movement**	**Fundus hypo-pigmentation**	**Fundus foveal hypoplasia**	**Iris hypo-pigmentation**
II:1	Male	7 y	Since birth	0.08/0.08 (corrected 0.3/0.3)	Conjugate, horizontal	No (obvious head tilt)	Yes	Yes	No
II:2	Male	1 y 11 m	Since birth	NA	Conjugate, horizontal	No	Yes	Yes	No
