# Supplementary material for: Deep intronic GPR143 mutation in a Japanese family with ocular albinism
Source: Sci Rep. 2015 Jun 10;5:11334. doi: 10.1038/srep11334 (PMC4650666; doi:10.1038/srep11334)
Supplement: Supplementary Information [file srep11334-s1.pdf]

Deep intronic *GPR143* mutation in a Japanese family with ocular albinism

Takuya Naruto[1], Nobuhiko Okamoto[2,*], Kiyoshi Masuda[3], Takao Endo[4], Yoshikazu Hatsukawa[5], Tomohiro Kohmoto[3,6], Issei Imoto[3,*]

[1] Department of Stress Science, Institute of Biomedical Sciences, Tokushima University Graduate School, Tokushima 770-8503, Japan

[2] Department of Medical Genetics, Osaka Medical Center and Research Institute for Maternal and Child Health, Izumi 594-1101, Japan

[3] Department of Human Genetics, Institute of Biomedical Sciences, Tokushima University Graduate School, Tokushima 770-8503, Japan

[4] Department of Ophthalmology, Osaka University Graduate School of Medicine, Suita 565-0871, Japan

[5] Eye Department, Osaka Medical Center and Research Institute for Maternal and Child Health, Izumi 594-1101, Japan

[6] Student Lab, Tokushima University Faculty of Medicine, Tokushima 770-8503, Japan

Supplementary information

Table S1. Candidate variants corresponding to X-linked recessive or autosomal recessive model, including compound heterozygosity observed in this family

Chr	Position	Gene		Ref	Alt	Mutation	Amino acid	Inheritance	Prediction of functional damage			Additional frequency data	
		symbol	Locus						SIFT[a]	Polyphen2[b]	MutationTaster[c]	1000Genome[d]	HGVD[e]
16	450,140	*NME4*	NM_001286435	-	AG	insertion	R158fs	AR	NA	NA	NA	(-)	(-)
19	37,879,852	*ZNF527*	NM_032453	-	TGTG	insertion	P301fs	AR	NA	NA	NA	(-)	(-)
X	36,397,596	*CXorf30*	NM_001098843	T	G	aTg/aGg	M552R	XR	0.01	0.999	0.6830	(-)	0.008
X	32,613,957	*DMD*	NM_000109	C	T	Gtc/Atc	V499I	XR	0.09	0.997	1.0000	(-)	0.003

NA, not available

[a]http://www.blocks.fhcrc.org/sift/SIFT.html

[b]http://genetics.bwh.harvard.edu/pph2/

[c]http://www.mutationtaster.org/

[d]1000 Genomes Project 2012 April data sets (http://www.openbioinformatics.org/annovar/)

[e]The Human Genetic Variation Database, a reference database of genetic variations in Japanese population from the Japanese Genetic Variation Consortium (http://www.genome.med.kyoto-u.ac.jp/SnpDB)

Table S2. Summary of the performance of whole-exome sequencing analysis

Family members		Alignment reads[a]	Mapped sequence (Gb)	Target bases				Filter-passed variants[b]	
Sample	Status			>1x (%)	>5X (%)	>20x (%)	Mean coverage	SNV	Indel
II:1	Affected	86,662,144	3.0	99.0	97.9	93.3	97.0	44,542	3,399
II:2	Affected	87,408,458	3.1	99.1	97.9	93.5	100.3	43,651	4,129
I:1	Unaffected	178,512,332	5.6	99.4	98.5	96.7	180.0	44,351	4,318
I:2	Unaffected	88,468,340	3.2	99.0	97.8	93.6	101.7	44,450	3,489

[a]PCR duplicates were removed

[b]Variants that passed GATK were filtered manually

Table S3. Primers used for direct sequencing, minigene construction and RT-PCR

**GPR143 intron 5 (mutation detection)**	
GPR143 intron 5_Forward	TAGTTTCTGAACTAGGAGCATTCA
GPR143 intron 5_Reverse	CCGTGTAAATGCCTTGTCTTCC
**GPR143 intron 5 and flanking exonic sequences (minigene construction)**	
Minigene_Forward_XhoI	AAAACTCGAGCTGTGGTGACCAGTGACTCC
Minigene_Reverse_NotI	AAAAGCGGCCGCTCAAAGGGCACCTAGCACAG
**RT-PCR to detect GPR143 exon 5-6 transcript**	
GPR143 exon 5-6_Forward	CCCCACTATGTCACCATGTACC
GPR143 exon 5-6_Reverse	CGTTCTCCGTGTAAATGCCTTG
